# Psychometric Analysis of the Czech Version of the Toronto Empathy Questionnaire

**DOI:** 10.3390/ijerph18105343

**Published:** 2021-05-17

**Authors:** Lukas Novak, Klara Malinakova, Petr Mikoska, Jitse P. van Dijk, Filip Dechterenko, Radek Ptacek, Peter Tavel

**Affiliations:** 1 Olomouc University Social Health Institute, Palacky University in Olomouc, 771 11 Olomouc, Czech Republic; klara.malinakova@oushi.upol.cz (K.M.); petr.mikoska@oushi.upol.cz (P.M.); j.p.van.dijk@umcg.nl (J.P.v.D.); peter.tavel@oushi.upol.cz (P.T.); 2 Department of Community and Occupational Medicine, University Medical Center Groningen, University of Groningen, 9713 AV Groningen, The Netherlands; 3 Graduate School Kosice Institute for Society and Health, P.J. Safarik University in Kosice, 040 11 Kosice, Slovakia; 4 College of Polytechnics Jihlava, 586 01 Jihlava, Czech Republic; Filip.Dechterenko@vspj.cz; 5 Department of Psychiatry, First Faculty of Medicine, Charles University in Prague and General University Hospital in Prague, 128 00 Prague, Czech Republic; ptacek@neuro.cz

**Keywords:** TEQ, empathy, validation, negatively worded items, psychometric examination

## Abstract

Empathy is a concept associated with various positive outcomes. However, to measure such a multifaceted concept, valid and reliable tools are needed. Negatively worded items (NWIs) are suspected to decrease some psychometric parameters of assessment instruments, which complicates the research of empathy. Therefore, the aim of this study was to assess the factor structure and validity of the TEQ on the Czech population, including the influence of the NWIs. Data were collected from three surveys. In total, 2239 Czech participants were included in our study. Along with socio-demographic information, we measured empathy, neuroticism, spirituality, self-esteem, compassion and social desirability. NWI in general yielded low communalities, factor loadings and decreased internal consistency. Therefore, in the next steps, we tested the model consisting of their positively reformulated versions. A higher empathy was found in females, married and religious individuals. We further found positive associations between empathy, compassion and spirituality. After the sample was split in half, exploratory factor analysis of the model with reformulated items was followed by confirmatory factor analysis (CFA), which supported a unidimensional solution with good internal consistency: Cronbach’s α = 0.85 and McDonald’s ω = 0.85. The CFA indicated an acceptable fit χ^2^ (14) = 83.630; *p* < 0.001; CFI = 0.997; TLI = 0.995; RMSEA = 0.070; SRMR = 0.037. The Czech version of the TEQ is a valid and reliable tool for the assessment of empathy. The use of NWIs in Czech or in a similar language environment seems to be questionable and their rewording may represent a more reliable approach.

## 1. Introduction

The concept of empathy has received increasing research attention in recent years. Although this concept can be defined variously, in general, it reflects “the drive or ability to attribute mental states to another person/animal, and entails an appropriate affective response in the observer to the other person’s mental state” [1]. From a more detailed view, it is thought that empathy has two facets. Emotional empathy reflects an emotional reaction (e.g., joy) to the feelings of others [2] while cognitive empathy is “cognitive and intellectual apprehension of another person’s emotional state” [3].

Empathy (both of its facets) is a key factor that allows successful social interaction [2]. For instance, some developmental disorders (e.g., autism spectrum disorder) characterized by problems in social functioning are also linked to decreased cognitive [4] and affective empathy [5]. However, this capacity is not only related to successful social interchange, but it also provides important benefits for professions in which working with people is a central characteristic. For instance, it has been shown that a higher level of empathy of medical professionals is associated with higher satisfaction with health care [6,7,8], with more frequent adherence to treatment [7], or with lower complaints from patients [9]. The empathic abilities of medical professionals were also associated with lower anxiety [7] and distress in patients suffering from an oncological disease [8]. Moreover, empathy and associated concepts (such as compassion) seem to be linked with a lower probability of developing burnout in medical professionals [10,11]. Empathy is also a crucial ability for workers in the fields of management [12], education [13], sales [14] and in psychotherapy [11,15].

A number of empathy self-report measures are validated in the English language, allowing one to explore the benefits of empathy. Some years ago, the Toronto Empathy Questionnaire (TEQ) was developed [2]. This new self-report instrument measures primarily the emotional aspects of empathy. Compared to some already developed measures, the TEQ has the following advantages: (1) it reflects more contemporary theories of empathy as it does not assess aesthetic projection [16] or personal distress—which seems to be rather related to neurotic traits than to empathy [2]; (2) it can be used in a wide range of research and practical contexts as it measures empathy towards people in general and not only to clinical practitioners or specific socio-cultural groups; (3) it is relatively brief and is thus more appropriate for use in larger test batteries. This unidimensional measure yields a high internal consistency (Cronbach’s alpha > 0.85) and a high test-retest reliability (*r* = 0.87) [2]. Its validity was supported via positive correlation with the Empathy Quotient [1] (*r* = 0.80), the Reading the Mind in the Eyes test [17] (r = 0.35) and via negative correlation with the Autism Quotient [18] (*r* = −0.30) [2]. To date, the TEQ as validated also in Turkey [19], China [3] or Korea [20].

The TEQ consists of positively and negatively worded items (NWIs). Regarding the NWIs, there is an ongoing debate about their inclusion into assessment tools in general. Whereas some researchers argue that these items may reduce the agreement bias and improve scale validity [21], others provide evidence suggesting that they decrease the psychometric quality of items, e.g., discriminatory power and decrease in internal consistency of a scale [22]. However, in some measures that were psychometrically tested in the English language, in which NWIs were included, internal consistency was still relatively high, as well as factor loadings (FL) [2]. Therefore, in the English language, the use of NWIs does not always seem to lead to a large decline in internal consistency or FL.

However, previous validation studies [23,24,25,26], examining psychometric properties of various measures of Czech versions, revealed that NWIs are frequently associated with both decreased internal consistency of a scale and FL. It is therefore possible that in similar language environments (e.g., Slovak or Polish), NWIs may influence the psychometric quality of an instrument in a similar way. This might have an important implication for studies validating new measures in these language environments. Reformulation of NWIs into positively worded items might increase some psychometric parameters of an assessment tool, while not forcing researchers to exclude inadequate items from a scale.

Along with testing the NWIs, we aimed to examine the psychometric properties of the Czech version of the TEQ in general. Such an examination can have important theoretical implications. An inherent feature of some psychometric studies is that they are testing a hypothesis about the theoretically driven structure of an instrument. In this sense, they either support or weaken a theory about some phenomena. Applied to the context of this study, by evaluating psychometric parameters of the TEQ in the Czech cultural environment, the specific empathy theory (in terms of theoretical structure) can be tested. This might in turn increase the general understanding of the empathy construct as conceptualized by the authors of the TEQ. Examining the psychometric properties involved convergent and divergent validity testing. Convergent validity was assessed by an association of the TEQ with gender, social desirability, compassion, and spirituality. Divergent validity was evaluated by examining the link between the TEQ, neuroticism, and self-esteem.

Thus, the aim of this study was to: (i) examine the psychometric properties of the Czech version of the TEQ in the Czech environment; (ii) evaluate the psychometric parameters of negatively worded items on the internal consistency and factor loadings; (iii) evaluate psychometric properties of positively reformulated negative items; (iiii) examine temporal stability of the TEQ score. These aims were realized in three consecutive studies: study 1 was conducted to pursue the first three aims (i,ii,iii), study 2 to pursue the first and the third aim (i,iii). Study 3 was designed to go with the last one (iiii). In total, eight hypotheses related to our study aim were formulated. A link to pre-registration of our hypotheses and a link to other Supplementary Materials (e.g., rationale for our hypotheses) can be found in the Supplemental Materials section. The specific hypotheses for study 1 can be found at the end of the introduction section. The two hypotheses from the pre-registration protocol were reformulated and their current wording can be found below (H7, H8).

**Hypothesis** **1 (H1).** *Alternative factor model of the Toronto Empathy Questionnaire (TEQ) explains the data better than the originally suggested one-dimensional model.*

**Hypothesis** **2 (H2).** *Compared to reformulated, positively worded TEQ items, items containing double negatives decreases the internal consistency of the scale.*

**Hypothesis** **3 (H3).** *There is a positive association between TEQ score and social desirability.*

**Hypothesis** **4 (H4).** *Empathy score is positively associated with compassion.*

**Hypothesis** **5 (H5).** *There is a positive correlation between empathy and spirituality.*

**Hypothesis** **6 (H6).** *There is a significant difference in empathy between males and females.*

**Hypothesis** **7 (H7).** *Trait neuroticism is positively associated with empathy.*

**Hypothesis** **8 (H8).** *Empathy is positively associated with self-esteem.*

## 2. Methods

### 2.1. Study Sample

Data in our study were acquired through the Olomouc University Social Health Institute (OUSHI) survey. The study was approved by the Ethical Committee of the Faculty of Theology, Palacky University Olomouc (No. 10/2018). Participation in the survey was fully voluntary, so the respondents could stop responding in the survey at any time. Subjects had to confirm that they read the informed consent before the survey beginning. Participants were not recruited from a neurological or psychiatric population. Prior to data analysis, this study was preregistered [27] in the Open Science Framework (OSF).

#### 2.1.1. Study 1

The data collection was done in two waves between September 2018 and January 2020. In both waves, data were acquired through snowball technique by both a paper–pen method and through an online questionnaire (78%: online; 22% paper–pen). In total, we collected data from 1141 participants (Age: *M* = 29.6; *SD* = 11.73; 68.5% females). Almost half of the participants (43.7%) were non-religious. In the unidimensional solution, there was no difference in the TEQ score between data collection waves or between an online survey and a paper–pen method. More detailed socio-demographic characteristics of the study sample are presented in Supplementary Table S1.

For further details regarding the missing data analysis, outliers screening, distribution of our data and estimation of the sample size for Confirmatory Factor Analysis (CFA) and Exploratory Factor Analysis (EFA), see Supplement one.

#### 2.1.2. Study 2

Subjects of the second sample were recruited in April and May 2020 by a specialized agency (i.e., the Czech National Panel) recruiting participants for research. All participants completed the survey online. The participants (*n* = 1036, Age: *M* = 49.3, *SD* = 16.55) were proportionally balanced in gender (50.7% females). More than one-half were non-religious (53.2%). Most of the participants were employed (47.5%).

#### 2.1.3. Study 3

From the retest study sample (Age: *M* = 25.4, *SD* = 8.93, *n* = 28, 79.2% females), most of the participants were students (75.0%), were not in any relationship (79.2%), completed secondary school with graduation (70.8%) and were non-religious (45.8%). Data from all participants were collected via an online questionnaire and by a snowball technique.

### 2.2. Measures

In studies 2 and 3, only one measure, i.e., the TEQ, was used. In study 1, the following measures were used:

#### 2.2.1. Toronto Empathy Questionnaire (TEQ)

The TEQ was developed to assess the general empathy factor or the “broadest level of empathy” with an emphasis on the emotional component, see [2]. This questionnaire consists of 16 items (half of them negatively worded) rated on a five-point Likert scale from “Never” (0) to “Always” (4). A higher score indicates higher empathy. Permission for translation and adaptation of the TEQ has been obtained from the scale author (Spreng). The translation process was based on the recommendations of WHO [28]—see Supplement one. We have reformulated the NWIs and administered the original negative items as well as their positively worded variants.

#### 2.2.2. Daily Spiritual Experience Scale (DSES)

The DSES [29] is a tool developed to measure an experience of connectedness to a transcendental sphere in everyday life [30]. The Czech version validated by Malinakova et al. [31] consists of 15 items rated on a 6-point scale ranging from “Many times a day” (1) to “Never or almost never” (6). A higher score indicates a higher degree of spirituality [32]. In this study, Cronbach’s α was 0.96.

#### 2.2.3. Santa Clara Brief Compassion Scale (SCBCS)

The SCBCS [33] is a measure assessing compassion tendency towards strangers [34]. The scale contains five items rated on a seven-point Likert scale ranging from “Not at all true of me” (1) to “Very true of me” (7). A higher score means a higher level of compassion. The Czech version was validated by Novak et al. [35] In this study, Cronbach’s α was 0.84.

#### 2.2.4. Rosenberg Self-Esteem Scale (RSES)

The RSES [36] is the most widely used self-esteem scale [37]. It consists of five positive and five negative statements about oneself, rated on the four-point Likert ranging from “Strongly Agree” (0) to “Strongly Disagree” (3) [37]. A higher score is indicative of higher self-esteem. In this study, Cronbach’s α was 0.83. The Czech version used in this study was validated by Blatný et al. [38]

#### 2.2.5. Short Social Desirability Scale (SDRS)

The SDRS [39] consists of five statements on a five-point Likert scale ranging from “Definitely true” (1) to “Definitely false” (4). Only extreme responses are scored. A higher score is indicative of a higher degree of social desirability. In this study, Cronbach’s α was 0.52. The mean inter-item correlation coefficient (MIIC) assessing the degree to which the score from a single item is related to the scores from all other scale items [40] resulted in the value of 0.18. According to Clark and Watson [41] and Parker et al. [42], the MIIC should not be less than 0.15.

#### 2.2.6. Big Five Inventory-Neuroticism Subscale (BFI-N)

The BFI is one of the most widely used personality trait measures [43]. Its neuroticism subscale assesses a tendency to experience feelings of anxiety, sadness or tension. This subscale consists of eight statements rated on a five-point scale ranging from “Strongly disagree” (1) to “Strongly agree” (5). A higher score indicates a higher degree of neuroticism. In this study, Cronbach’s α was 0.83. The Czech version was validated by Hrebickova et al. [44].

### 2.3. Procedure

Positively worded items were developed by just the positive reformulation of negative items. For instance, item 2 “Other people’s misfortunes do not disturb me a great deal” was positively reworded by changing the terms “do not” to “do”. Thus, the reworded variant of this item was: “Other people’s misfortunes disturb me a great deal”. In the next step, these reformulated items were added into a larger test battery (study 1) in which the original negatively worded items were also present. In studies two and three, only the TEQ with reformulated items was used.

### 2.4. Statistical Analysis

#### 2.4.1. Study 1

##### Outlier Detection, Missing Data

For the detection of outliers, we used a median absolute deviation (MAD). As recommended by Leys et al., [45] we treated values as outlying if they lied above 2.5 times of MAD or below—2.5 times of MAD. The MAD test was performed in R programming software [46] in the Routliers [47] package. Cases in which both univariate outliers have been found were complexly screened. This screening aimed to detect a uniform pattern of responding (i.e., the answer was the same in several consequent questionnaires), multiple logical incongruences (i.e., reporting atheism while also reporting feeling God’s presence many times a day) or other information suggesting that these cases should be excluded from the analysis. If the presence of any of these problems would be observed (*n* = 0), the case would be removed from the dataset.

To explore the missing data pattern, we performed the Little MCAR test in the package BaylorEdPsych [48] in R. Results of the MCAR test for the TEQ items strongly suggest that data are missing completely at random χ^2^ (284) = 312.84, *p* = 0.11. Additionally, in those participants where missing values were detected (*n* = 67), the highest percentage of missing values was 27%. Due to a very low number of missing values in the data and due to the relatively large sample size, we have decided to exclude missing values listwise during statistical analysis.

##### Estimating the Number of Factors in the Data

For determination of a number of factors in the data, multiple criteria were used: (1) assessment of the adequacy of the factor model based on a theoretical coherence and parsimony; (2) the Kaiser criterion [49] together with non-parametric bootstrapping to produce confidence intervals for eigenvalues; (3) Scree plot test [50]; (4) Hull method [51]; (5) Parallel analysis (PA) [52]; (6) comparison data method (CD) [53] and (7) the Empirical Kaiser Criterion (EKC) estimating the number of factors based on Marchenko–Pastur distribution of eigenvalues [54].

##### Fitting Algorithm in EFA, Item Retention Rules and Replicability Index

The sample was randomly split in half; on one half, EFA was conducted, and CFA on the other. Factor models in the EFA were fitted by using the Weighted Least Squares (WLS) method. Across conducted EFAs, we focused on problematic items, which exhibited in the following order: (1) absence of factor loading; (2) theoretically incoherent factor loading; (3) insufficient h2; (4) low factor loading; and (5) cross-loading. Due to the character of the empathy construct, we presumed that extracted dimensions will be correlated. Thus, Oblimin rotation was used across all EFAs, in which a multidimensional structure was assumed. In case of unidimensional solutions, we used the Varimax rotation. The final factor solution was chosen based on the interpretability of the results. All correlation analyses were done on a polychoric correlation matrix in the psych package [55] in R.

Final factor solutions were evaluated for strength and replicability by H-Index [56]. The H-index values < 0.80 suggest that a latent variable is poorly defined and is probably unstable [57].

##### Confirmatory Factor Analysis (CFA)

The CFA was conducted in the R package lavaan [58]. The CFA models were fitted by the Diagonally Weighted Least Squares estimator (DWLS). The goodness-of-fit indexes being measured and an interpretation of their values can be also found in Supplement two.

##### Scale Homogeneity

Coherence of the scale items was assessed via the item-total correlation (ITC). The ITC was adequate if the correlation coefficient value was ≥0.40 [59,60]. Internal consistency of the scale was estimated via Cronbach’s alpha and McDonald’s ω [61]. The Cronbach’s α, the ITC and McDonald’s ω were calculated in the R packages psych and ufs [62].

#### 2.4.2. Study 2

Outliers were screened using the same procedure as in study 1. Missing values analysis was performed in the same way as in study 1 and indicated that values are missing completely on random. Thus, we excluded missing cases listwise during statistical analysis. The CFA was conducted in the R package lavaan [58] using the DWLS method.

#### 2.4.3. Study 3

Outliers were screened using the same procedure as in study 1. No missing data were present. The Intraclass Correlations (ICC) were used to assess the temporal stability of the TEQ scores one month after the first administration.

## 3. Results

### 3.1. Study 1: Factor Analysis, Convergent Validity and Reliability

#### 3.1.1. Comparison of the Original vs. Reformulated Items in EFA and Items Statistics

The TEQ items statistics (means, standard deviations, skewness, kurtosis, range) are reported in Supplement three. In the first EFA, we evaluated the potential influence of the NWIs by comparing them to their positively reworded analogues in two EFA models. The models from positive NWIs were consequently compared regarding their internal consistency, FL, h2 and an ITC.

The Bartlett’s test of sphericity indicated that data are sufficiently correlated to perform EFA: χ^2^ (210) = 9694.92, *p* < 0.001. Sampling adequacy assessed by Kaiser–Meyer-Olkin (KMO) measurements resulted in a mean sampling adequacy of 0.88, suggesting that factorability of data is good. The EFA was conducted based on 5000 bootstrapped samples using a varimax rotation and assuming a unidimensional (original) structure.

The EFA results indicated that FL was higher in all reworded items as compared to their original versions (Table 1). Regarding h2 of the original reverse items, all might be considered insufficient. However, when h2 was calculated in reworded items, h2 in most of the items increased to a sufficient level (Table 1).

The TEQ with the original items displayed lower internal consistency compared to the scale with the reworded items. The ITC suggested that the reworded items are more consistent with the scale (Table 1).

Taken together, assessed psychometric parameters of the reworded TEQ items were in general better, compared to their original variants (supporting our H2). Thus, for further analysis (with the exception of the first CFA), reworded items were used. In the next step, a series of EFAs was conducted. During these EFAs, we excluded items that were not in line with the item retention rules defined in the methods section. See Supplementary Table S2 for a detailed description of this procedure. This process resulted in one (General empathy) factor solution consisting of seven items (see Supplement four for details).

#### 3.1.2. Confirmatory Factor Analysis

##### The Original Model vs. the Model with Reworded Items

To evaluate the results of the first EFA, the original TEQ structure [2] based on all 16 original items (including negative) was explored. Results indicated that some items (TEQ 11) had substantially low or even a negative FL (TEQ 14). Moreover, compared to the original model, the scale with the reformulated items yielded a higher fit, lower residuals (Table 2) and in general a higher FL (thus supporting results of the EFA).

##### The Final CFA Model

The CFA results further indicated that the General empathy model seems to be an optimal compromise among different factor models. Therefore, we failed to reject our null hypothesis in H1. See Table 2 for the goodness-of-fit indexes of this model. The item composition of each model can be found in Table 3. For an in-depth analysis of the individual CFA factor models, see Supplement five.

#### 3.1.3. Reliability and Replicability

Reliability of the General empathy factor was satisfactory, with Cronbach’s α = 0.85, 95% CI [0.84–0.86] and McDonald’s ω = 0.85, 95% CI [0.84–0.86]. Moreover, the General empathy model seems to be stable and replicable (H = 0.87). The ITC of this model was acceptable (Table S4 in Supplement five). Correlation between individual items and item statistics of the General empathy factor can be found in Table 4.

#### 3.1.4. Convergent Validity

**Figure 1:**
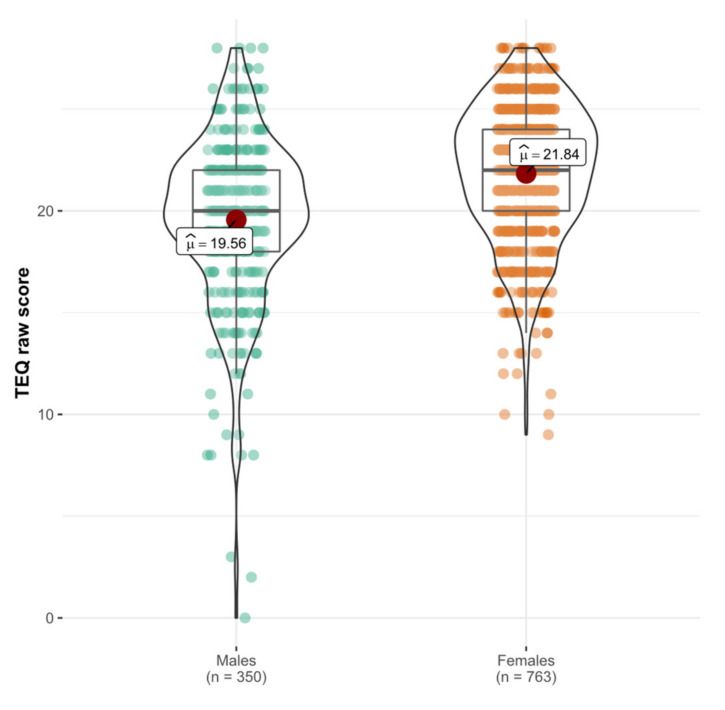
Violin plot with gender differences in empathy (Study 1, *n* = 1141). The TEQ score distribution across two genders; $\hat{\mu }$ = means empathy score. TEQ raw score is calculated from the final set of items resulting from the EFA. TEQ = Toronto Empathy Questionnaire.

Contrary to our third hypothesis (H3), the TEQ was not significantly associated with social desirability $r_{s}=0.12$, p=0.097. However, due to the low internal consistency of the social desirability measure, the results need to be interpreted with caution. Congruently with our H4, the TEQ was positively correlated with the trait compassion $r_{s}=0.61$, p<0.001. As expected (H5), the tendency to experience transcendental feelings was associated with the TEQ $r_{s}=0.23$, p<0.001. In line with our H6, the Welch T-test revealed a higher TEQ score in females with medium effect size $t\left(565.61\right)=-9.12$, p<0.001, *d* = 0.637, 95% CI [0.502–0.772] (see Figure 1). As the male group had outliers at the lower end of the distribution, we performed Yuen’s test [64] based on trimmed means to decrease the influence of outliers on results. Yuen’s test indicated a significant difference between genders in empathy t_Yuen_ (529.26) = 9.29, *p* < 0.001, Cohen’s delta = 0.43. In contrast with H7 and H8, a relationship between the TEQ and trait neuroticism ($r_{s}=-0.03$, p=0.764) and the RSES score ($r_{s}=0.09$, p=0.186) was non-significant.

### 3.2. Study 2: Replication of the Factor Structure

In order to check whether better psychometric characteristics (FL and h^2^) of the positively reformulated items were not influenced by the simultaneous presence of NWIs, we administered the TEQ without negatively worded items to a separate group of participants (*n* = 1036). See Supplement six for a detailed description of the study sample, measures and assumptions testing. Results of the CFA indicated that the General empathy model has a good incremental model fit and acceptable absolute fit χ^2^ (14) = 83.630; *p* < 0.001; CFI = 0.997; TLI = 0.995; RMSEA = 0.070 95% CI [0.056, 0.084]; SRMR = 0.037 (see Table 2 for meaning of abbreviations). Model fit indicators suggested a higher fit compared to the CFA on the previous sample. 

**Figure 2:**
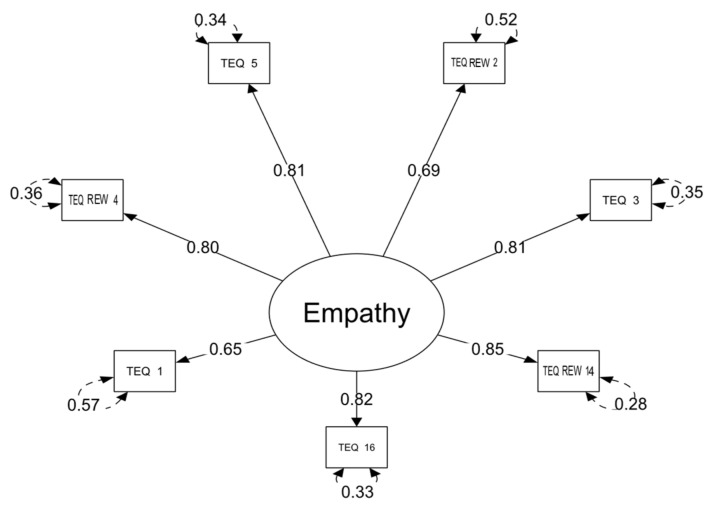
The General empathy model of TEQ with factor loadings and residuals (Study 2, *n* = 1036). The “REW” in item 2, 4 and 14 refers to positively reformulated negative items.

In the General empathy model, with one exception (item 2), the reworded items displayed higher FL and h^2^ in the CFA conducted on the second sample (Table 4) as compared to the CFA on the first sample. As expected, almost all items (except item 1) displayed low residuals and high factor loading (see Table 4 and Figure 2). Thus, the results of the CFA on the second sample supported the findings of the CFA conducted on the first sample.

### 3.3. Study 3: Test–Retest Reliability

In the last part of our study, we evaluated the test–retest reliability of the TEQ on a separate sample via ICC. More details about this retest study sample can be found in Supplement seven. The ICC indicated that stability of the TEQ scores was good *r* = 0.81, 95% CI [0.59–0.91], *p* < 0.001.

## 4. General Discussion

The aim of this study was to examine the psychometric properties of the Czech version of the Toronto Empathy Questionnaire (TEQ) in the Czech population and to explore the psychometric parameters of NWIs compared to their positively reformulated analogues. Results indicated that the one-factor model is a stable and replicable solution of the TEQ. It was also found that in general, NWIs yielded a lower psychometric quality, especially in terms of FL, h^2^ and reliability, as compared to their positively worded variants. Thus, we offer a new version of the TEQ consisting of only positively worded items. Validity testing indicated no significant association between TEQ score and social desirability. The TEQ was positively associated with compassion, spirituality and female gender; no significant associations were found with trait neuroticism and self-esteem.

Our results supported the original unidimensional factor structure [2]. These findings are, however, in contrast with the Korean version validated by Yeo and Kim [20] and with the Chinese version of the TEQ [3], which both suggested a three-factor structure. On the other hand, our results are in line with the Korean version of the TEQ validated by Hwan and Sumi [65], where a one-factor solution was also recommended. However, unlike the Hwan and Sumi [65], our goodness-of-fit measurements indicated a better model fit in both studies. One of the possible reasons why these authors achieved a lower model fit is that items with a relatively low FL were included in their final model. Indeed, the goodness of fit indexes were better in TEQ validation studies [13,19], where items with insufficient FL have been excluded.

We also found that the first TEQ item (“When someone else is feeling excited, I tend to get excited too”) had in both CFA studies the lowest FL and communalities of all items. Its different behaviour might be explained by the fact that it is the only item assessing emotional sharing. The rest of the items assess either emotional and behavioural responses towards people in need or general emotional reactions to the happiness of others. Furthermore, as compared to other studies [3,20] item 11 (“I become irritated when someone cries”) had an extremely low correlation with the total score as well as low factor loadings. On the other hand, authors of the Greek version of the TEQ [13] also excluded the item 11 due to its low psychometric parameters. As the reasons for its low properties are not clear, future studies should be conducted to explore why item 11 has a low measurement quality in the Czech version of the TEQ.

This study showed that positively reformulated items have adequate psychometric properties even when administered without original negatively worded items. This supports the results of the first study regarding better psychometric characteristics of positively reformulated items over negative items. This finding is congruent with the results of other studies, see [63]. There might be several explanations why NWIs display a decreased psychometric quality. A first reason could be that, compared to positively worded items, NWIs (especially double negatives) require a higher degree of language skills in order to be correctly understood [22]. It is therefore possible that in some participants, a lower language skill level could decrease the chance that they will understand and answer the items in the wrong way. Thus, NWIs seem, at least partially, to also measure language skills. Therefore, their use is questionable, especially in situations where empathy is assessed in individuals with decreased language skills. The second reason is that double negatives can be differently used in the Slavistics and in the English language environment (see [66,67]). More frequent use of double negatives in certain contexts by the native English speakers—as compared to Czechs—can indicate that English language natives are more used to speaking with these double negatives, which might consequently decrease the cognitive language skill demand, while increasing the chance that they will be correctly understood.

The strong positive relationship we found between empathy and compassion is in line with findings of studies that reported a strong [2,16] to moderate [68,69] correlation between these two constructs. As noted by Spreng et al. [2], empathy, as measured by the TEQ, is a very similar construct to compassion. This similarity can imply that some psychological processes can be shared between these two capacities. According to Goetz et al., [70] empathy processes form the necessary condition for compassionate experience to occur. Empathy in the sense of affect sharing is from this perspective viewed as an element eliciting the compassion. Therefore, our finding regarding the strong relationship between these two constructs can be explained by a strong interconnection of psychological processes between empathy and compassion.

An association between higher empathy scores and female gender is in line with the results of studies utilizing the TEQ to detect gender differences in empathy [13,16,17,63,65]. Higher empathy in females can be explained by several mechanisms, such as social expectations (i.e., society, in general, expects females to be more empathetic) [71], hormonal factors (i.e., different neuroendocrine functioning) [72] or possibly differences in brain structure [73]. One prominent explanation is the Primary Caretaker Hypothesis. This theory suggests that the evolution process favored the survival of infants, whose caregivers (typically females) were more sensitive towards their needs. Thus, the selection pressure may have stimulated the higher sensitivity to the needs of others predominantly in the female gender [74]. However, this explanation seems to contradict findings from studies with more sophisticated designs in which no significant difference in empathy between genders was detected [75].

We also did not find a significant association between empathy and self-esteem. This finding is in line with the conclusions of many other studies [76,77,78,79], while also contradicting others [80,81]. This contradiction can be explained by the different conceptualization of empathy. As indicated by Cone [76], across studies can be found a relatively stable relationship between self-esteem and cognitive empathy, while in the studies examining self-esteem in relation to emotional empathy, the results are usually not significant.

As expected, this study revealed that there is no relationship between neuroticism and the TEQ score, which is in line with findings of other studies [82,83,84]. Our results support the validity of the TEQ, as this scale was not designed to measure neurotic traits associated with sharing of emotional distress of other people. The absence of this relationship can be explained by a strong similarity between the construct being measured by the TEQ and compassion, which is unrelated to neurotic traits [85,86].

### 4.1. Strengths and Limitations

Our study has several strengths: first, a large sample size allowed us to perform EFA, CFA and CFA replication on different samples. This procedure makes our results robust and credible. Second, unlike many other validation studies of the TEQ, we used recently developed and reliable methods for the exploration of the latent structure of the instrument, which adds credibility to our factor analytic findings. Third, this study was preregistered and due to publicly accessible data and code, its results can be easily and fully replicated. Fourth, a new version of the tool based on positively worded items was developed and these positively worded items yielded better psychometric parameters as compared to NWIs.

However, our study also has some limitations. First, despite the fact that we did not find evidence for the effect of social desirability on the TEQ score, this effect cannot be ruled out. Second, the TEQ is a self-report measure and thus it suffers from limitations inherently related to such kinds of assessment tools. Third, we used the Czech version, and have not assessed a version in any other Slavonic language, which limits the generalizability of the findings on the whole Slavonic language environment. Fourth, we did not use the probabilistic sampling method, which further limits the generalizability of our findings. Finally, as the positively reworded items were administered in the same test battery as the original negatively worded items, the effect of order cannot be ruled out.

### 4.2. Implications

The Czech version of the TEQ could be used by practitioners when the aim is to assess the degree of general empathy, especially its affective component. The TEQ can be utilized in practice in several ways. First, if the norms are created, the TEQ can be used for clinical assessment. It may help the clinician in the evaluation of psychological profiles, and it can help with the interpretation of the results from other tests. Second, it can be used by working psychologists in the selection of employees in positions where the degree of general empathy or its emotional component strongly influences work outcomes, e.g., in nursing.

There might be several recommendations for future research. First, criterion-related validity of the Czech version of TEQ should be examined while using validated empathy measures. Second, future researchers might consider modification of the original scale and add more items measuring positive empathy. Lastly, studies examining the psychometric properties of assessment tools containing the negative items should take into account the fact that in different language environments, negatively worded items might be understood differently. At least in the Slavonic language environment, researchers might consider reformulating negative items into positive ones to increase some psychometric properties of instruments.

## 5. Conclusions

The results of this study indicate that the TEQ is a valid and reliable tool for assessing the General empathy factor in the Czech population. Future studies should explore the criterion validity of the instrument. Researchers validating new instruments, at least in the Slavonic language environment, should consider reformulating negatively worded items into positive.

## Figures and Tables

**Table 1:** Original and reworded item characteristics (Study 1, *n* = 1141).

				TEQ				
Original Items					Reformulated Items			
	Factor Loading	h^2^	ITC			Factor Loading	h^2^	ITC
TEQ_1	**0.60**	0.36	**0.54**		TEQ_1	**0.56**	0.31	**0.55**
TEQ_2	0.31	0.09	0.37		TEQ_REW_2	**0.72**	**0.51**	**0.69**
TEQ_3	**0.67**	**0.45**	**0.63**		TEQ_3	**0.70**	**0.49**	**0.70**
TEQ_4	**0.50**	0.25	**0.52**		TEQ_REW_4	**0.63**	0.40	**0.62**
TEQ_5	**0.66**	**0.44**	**0.57**		TEQ_5	**0.63**	0.40	**0.62**
TEQ_6	**0.57**	0.33	**0.52**		TEQ_REW_6	**0.66**	**0.44**	**0.64**
TEQ_7	0.37	0.13	**0.41**		TEQ_7	0.32	0.10	0.36
TEQ_8	**0.40**	0.16	0.39		TEQ_8	0.39	0.15	**0.41**
TEQ_9	**0.53**	0.28	0.55		TEQ_9	**0.51**	0.26	**0.55**
TEQ_10	0.31	0.09	0.35		TEQ_REW_10	**0.53**	0.28	**0.54**
TEQ_11	0.01	0.00	0.05		TEQ_11	−0.05	0.00	−0.03
TEQ_12	**0.58**	0.34	**0.60**		TEQ_REW_12	**0.75**	**0.56**	**0.77**
TEQ_13	−0.36	0.13	−0.18		TEQ_13	−0.29	0.08	−0.28
TEQ_14	−0.56	0.31	−0.39		TEQ_REW_14	**0.76**	**0.58**	**0.74**
TEQ_15	**0.46**	0.21	**0.58**		TEQ_15	**0.40**	0.16	**0.45**
TEQ_16	**0.65**	**0.42**	**0.59**		TEQ_16	**0.73**	**0.53**	**0.71**
Cronbach’s α	0.71 95% CI [0.68–0.74]				Cronbach’s α	0.84 95% CI [0.82–0.86]		
McDonald’s ω	0.73 95% CI [0.69–0.76]				McDonald’s ω	0.85 95% CI [0.84–0.87]		

Note. ITC: inter—total correlation corrected for item overlap and scale reliability (bold values ≥ 0.40); h^2^: communalities (bold values > 0.40); bolded values in FL ≥ 0.40; TEQ_REW: reworded item; TEQ: Toronto empathy questionnaire, CI: confidence interval.

**Table 2:** Model fit and residual indexes of CFA models (Study 1, *n* = 1141).

	CFA Models						
Fit and Residual Indexes	Original TEQ	Reformulated TEQ	Two Correlated Factors—Chiorri [63]	Two Correlated Factors—EFA	Hierarchical	General Empathy	Bi-Factor
χ^2^	625.152	457.919	613.168	28.869	28.869	56.453	15.312
df	104	104	103	13	12	14	3
p-value	0.000	0.000	0.000	0.007	0.004	0.000	0.002
CFI	0.897	0.963	0.900	0.996	0.995	0.988	0.994
TLI	0.882	0.957	0.883	0.993	0.992	0.982	0.968
SRMR	0.081	0.074	0.080	0.039	0.039	0.057	0.034
RMSEA	0.097	0.080	0.097	0.046	0.050	0.073	0.085
RMSEA 90% CI	0.090–0.105	0.073–0.087	0.089–0.104	0.023–0.069	0.027–0.073	0.054–0.94	0.046–0.129
χ^2^/df	6.01	4.4	5.95	2.22	2.41	4.03	5.1

Note. CFI: Confirmatory Factor Analysis, TEQ: Toronto Empathy Questionnaire, EFA: Exploratory Factor Analysis, χ^2^: Chi-Square, df = degrees of freedom, CFI: Comparative Fit Index, TLI: Tucker Lewis index, RMSEA: Root Mean Square Error of Approximation, SRMR: Standardized Root Mean Square Residual.

**Table 3:** Item composition of the tested models.

Models	TEQ Items																					
-	1	2	3	4	5	6	7	8	9	10	11	12	13	14	15	16	2R	4R	6R	10R	12R	14R
Original model	GE	GE	GE	GE	GE	GE	GE	GE	GE	GE	GE	GE	GE	GE	GE	GE						
Original with R items	GE		GE		GE		GE	GE	GE		GE		GE		GE	GE	GE	GE	GE	GE	GE	GE
Chiorri model [63]	E	C	E	C	E	E	C	E	E	C	C	C	E	C	C	E						
EFA model	PE		NE		PE											NE	NE	PE				NE
Hierarchical model	PE, GE		NE, GE		PE, GE											NE, GE	NE, GE	PE, GE				NE, GE
General Empathy model	GE		GE		GE											GE	GE	GE				GE

Note. R = reformulated item, EFA = Exploratory Factor Analysis, GE = General Empathy, E = Empathy dimension, C = Callousness dimension, NE = Negative empathy subscale, PE = Positive empathy subscale.

**Table 4:** Factor Loadings and communalities in two CFA studies.

	Study 1 (*n* = 1141)		Study 2 (*n* = 1036)	
Manifest variables	FL	h^2^	FL	h^2^
TEQ 1	0.52	0.27	0.65	0.43
TEQ REW 2	0.79	0.63	0.69	0.48
TEQ 3	0.75	0.56	0.81	0.65
TEQ REW 4	0.54	0.29	0.80	0.64
TEQ 5	0.60	0.37	0.81	0.66
TEQ REW 14	0.75	0.57	0.85	0.72
TEQ 16	0.71	0.50	0.82	0.67

REW = positively reworded item, FL = Factor Loadings, h^2^ = communalities.

## Data Availability

The data presented in this study are openly available in Open Science Framework at https://doi.org/10.17605/OSF.IO/8UX46, reference number 8UX46.

## Supplementary Materials

The following are available online at https://www.mdpi.com/article/10.3390/ijerph18105343/s1. Code used for statistical analysis, rationale for the study hypotheses and other materials can be found on the Open Science Framework website [accessed date: 12 May 2021] (https://osf.io/z85cv/). Pre-registration protocol can be found under the following link: https://osf.io/dnt8q.
