# Supplementary material for: Psychometric Analysis of the Czech Version of the Toronto Empathy Questionnaire
Source: Int J Environ Res Public Health. 2021 May 17;18(10):5343. doi: 10.3390/ijerph18105343 (PMC8156475; doi:10.3390/ijerph18105343)
Supplement: Supplementary file 1 [file ijerph-18-05343-s001.zip › Supplementary table 1.pdf]

**Supplementary Table S1:** Socio-demographic results

| Socio-demographic variables | Variable | n (%) | Empathy score | | |
|---|---|---|---|---|---|
| | | | $25^{th}$ percentile | M (SD) | $75^{th}$ percentile |
| Gender | Male | 353 (31.5%) | 18.00 | 19.6 (4.1) | 22.00 |
| | Female | 767 (68.5%) | 20.00 | 21.8 (3.3) | 24.00 |
| Family status | In relationship | 462 (41.8%) | 19.00 | 21.1 (3.5) | 24.00 |
| | No relationship | 333 (30.1%) | 18.00 | 20.2 (4.2) | 23.00 |
| | Married | 246 (22.2%) | 20.00 | 22.0 (3.2) | 25.00 |
| | Divorced | 28 (2.53%) | 20.75 | 22.6 (3.7) | 25.00 |
| | Widow | 18 (1.63%) | 17.25 | 20.2 (4.5) | 24.00 |
| | Friar | 19 (1.72%) | 19.50 | 21.9 (3.6) | 25.00 |
| Education | Basic school | 73 (6.41%) | 18.00 | 20.4 (4.6) | 24.00 |
| | Vocational school/high school without a graduation exam | 55 (4.83%) | 19.00 | 21.9 (3.9) | 25.00 |
| | High school | 490 (43.1%) | 19.00 | 21.0 (3.6) | 24.00 |
| | Higher vocational school | 43 (3.78%) | 19.00 | 21.4 (3.2) | 24.00 |
| | University unspecified | 345 (30.3%) | 19.00 | 21.1 (3.6) | 24.00 |
| | University bachelor | 29 (2.55%) | 20.00 | 20.3 (6.1) | 23.00 |
| | University master or Dr | 103 (9.05%) | 19.00 | 21.6 (3.2) | 24.00 |
| Economical status | Student | 384 (36.1%) | 19.00 | 21.1 (3.7) | 24.00 |
| | Invalidity pensioner | 10 (0.94%) | 22.00 | 21.7 (7.8) | 25.00 |
| | Employed | 451 (42.3%) | 19.00 | 21.2 (3.5) | 24.00 |
| | Entrepreneur | 80 (7.51%) | 19.00 | 20.8 (4.5) | 24.00 |
| | In household | 17 (1.60%) | 18.00 | 20.4 (3.0) | 23.00 |
| | Without work | 15 (1.41%) | 17.50 | 19.1 (3.1) | 21.00 |
| | Pensioner | 28 (2.63%) | 16.75 | 20.8 (4.2) | 24.25 |
| | Maternity leave | 80 (7.51%) | 18.75 | 20.8 (4.1) | 24.00 |
| Religiosity | Religious, member of a church | 286 (25.2%) | 20.00 | 21.8 (3.3) | 24.00 |
| | Religious, not a member of a church | 256 (22.6%) | 19.00 | 21.7 (3.3) | 24.00 |
| | Non-religious | 497 (43.9%) | 18.50 | 20.7 (4.0) | 23.00 |
| | Atheist | 94 (8.30%) | 17.00 | 19.3 (4.3) | 22.00 |

*Note.* M = mean, SD = Standard Deviation, *n* = number of subjects
