# Supplementary material for: Psychometric Analysis of the Czech Version of the Toronto Empathy Questionnaire
Source: Int J Environ Res Public Health. 2021 May 17;18(10):5343. doi: 10.3390/ijerph18105343 (PMC8156475; doi:10.3390/ijerph18105343)
Supplement: Supplementary file 1 [file ijerph-18-05343-s001.zip › Supplements (1-7).pdf]

Supplements (1-7)

Supplement one:

1.1. Translation of the instrument

Two fluently English-speaking psychologists independently translated the original version of the TEQ to the Czech language. After a backward translation, an expert panel was formed. Incongruences between backward translation and original TEQ items were discussed in this panel and based on this discussion, the final version of the TEQ was created. Another result of this discussion was an addition of four positively reworded items which were in the next step (qualitative interviews) administered together with the original 16-items. These interviews indicated that although some participants reported difficulties with responding to negatively worded items, overall, the TEQ items were comprehensible and the participants understood them in desired way.

1.2. Sample of the first study

Data collection among a Czech adult population was conducted in two waves. During the first wave, the data were collected primarily from September to December 2018 from three sources: Firstly, from Facebook (≈ 61%), where respondents filled an online questionnaire. Another part (≈ 7%) of the data was collected by snowball technique and pen and paper method. The second wave of data collection took place from September 2019 to January 2020. As in the first wave, data (≈ 17%) were collected using the online questionnaire. A small part (≈ 5%) of our data comes from another study. Another source of data (≈ 10%) in the second wave consists of questionnaires filled by the university students by pen and paper method. In both data collection waves, the respondents had no time limit to finish the questionnaire

1.3. Estimation of sample size for EFA and CFA

In line with the recommendations of Watkins [1], we report rationale regarding sample size estimation for EFA. We approximated the number of participants for EFA based on the estimation of sample for the goodness of fit indexes. We firstly calculated the number of participants needed to reach the desired level of accuracy in one of the absolute model fit measures. This calculation was done in the *MBESS* package in R [2]. Results indicated that the desired accuracy can be achieved with at least 699 participants.

Although this result is indicative primary for CFA, it can be indirectly used for estimation of sample size for EFA. We decided to split-half our data, and on the one half, we performed EFA (calibration sample) whereas on another half we conducted CFA (validation sample).

1.4. Missing data analysis:

We collected 1172 records. From these records, we deleted 20 records that contained only basic sociodemographic information while missing all other variables. In addition, we deleted 11 records in which neither of the TEQ items were answered. This resulted in 1141 records. The records were further evaluated for the number of missing data. We found that in 70 records there was one or more missing value in the TEQ items.

1.5. Outlier detection and exclusion:

In line with the methodological recommendations [3], we provide a description, how we detected and removed outliers. Based on the Median Absolute Deviation (MAD), we detected 18 outliers in the TEQ records. Results from outliers screening test using MAD and subsequent screening of records in which data were outlying did not suggest a uniform pattern of responding or other information, indicating that record should be excluded from further analysis. Only one value in the variable age suggested misunderstanding of the

instruction and it was reverted to standard fashion. However, in the TEQ data, we did not find a systematic pattern of responding and/or multiple logical incongruences. Thus, we have decided to keep values suggested as outliers in our dataset.

1.6. Distribution of the data

The normality of the distribution was examined via visual inspection of histograms and the Mardia test [4] in the *MVN* package in R [5]. Inspection of the histograms suggested possible problems with skewnesses - items: 3,4,5,7,13 and 4 (reformulated). The curve of the TEQ_13 was strongly suggesting a floor effect. Mardia's test indicated that multivariate normality can be rejected (standardized multivariate skewness coefficient = 7070.27, $p < .001$; standardized multivariate kurtosis coefficient = 51.94, $p < .01$).

Because our data did not meet the multivariate normality assumption, further analyses were conducted with non-paramedic tests. However, we also performed the same analysis with parametric methods, and if results from both methods were similar, then we reported results from parametric tests. Homoscedasticity and linearity assumptions were examined via visual inspection of the plots. Such visual inspection did not suggest serious heterogeneity of variances or non-linearity.

1.7. Exploratory factor analysis

1.7.1. Factor extraction

For our decision regarding how many factors to retain, the most important were Hull method, PA, and CD which can together provide an accurate estimation of dimensionality large sample sizes (≥ 500) [6].

1.7.2. Extraction algorithm method and EFA item statistic

Concerning the method used for factor extraction, we have denied the possibility to use maximum likelihood (ML) method and related goodness of fit indexes for the determination of the number of factors, because accurate estimation of the ML method is conditioned by multivariate normality [1,7] which was not met in our data. Thus, Instead of the ML method, our EFA factor models were fitted by the Weighted Least Squares method.

1.7.3. Item retention rules in the EFA

Communalities (h^2) for an individual items > .40 were considered as sufficient [8]. During the EFA procedure, items were representative of factor if:

a) factor loading ≥ .40

b) if cross-loading were not > .30

c) 3 ≥ variables per factor

d) alpha reliability for an individual factor ≥ .70 [1,9,10].

2. Supplement two

2.1. Confirmatory factor analysis – extraction algorithm (both studies)

The Root Mean Square Error of Approximation (RMSEA) and the Standardised Root Mean Square Residual (SRMR) examined an absolute model fit with the following values considered acceptable: the RMSEA ≤ 0.08 [11], the SRMR ≤ 0.08 [11]. The relative fit was examined with the Tucker-Lewis index (TLI) and comparative fit index (CFI) with TLI and CFI ≥ .95, suggesting an acceptable fit [12] and ≥ .97 a good fit [13]. Due to the multifaceted character of the empathy construct, the covariances among latent variables were estimated with no orthogonal restriction.

As the multivariate normality assumption of the TEQ items was broken in studies1 and 2, a nested model analysis could incorrectly reject the correct model [14]. Therefore, it was decided to use the χ^2 test developed by Satorra and Bentler, which is robust to violations of such assumption [15].

3. Supplement three

3.1. General psychometric characteristics of the TEQ items – item statistics

Results indicated that in several items, participants responses were situated either at the top of the scale (TEQ_3, TEQ_4, TEQ_5, TEQ_7, TEQ_15, TEQ_CON_4) (i.e. $M > 3$) or at its bottom (TEQ_13) (i.e. $M < 1$); see Table 1. In both cases, problematic items should be considered for exclusion from the scale [16]. Therefore, if factor loadings and communalities will be low in these problematic items, they will be excluded from the scale.

Table 1: *TEQ items descriptive statistic*

Items	*M*	*SD*	*Skewness*	*Kurtosis*	*min*	*max*
TEQ_1	2.64	0.72	-0.52	0.54	0	4
TEQ_2	2.33	0.90	-0.29	-0.22	0	4
TEQ_3	3.35	0.73	-1.10	1.42	0	4
TEQ_4	3.22	0.85	-1.24	1.71	0	4
TEQ_5	3.32	0.76	-1.24	2.27	0	4
TEQ_6	2.27	0.90	-0.14	-0.28	0	4
TEQ_7	3.35	0.77	-1.16	1.34	0	4
TEQ_8	2.91	0.70	-0.55	0.95	0	4
TEQ_9	2.66	0.83	-0.70	0.58	0	4
TEQ_10	2.43	1.04	-0.30	-0.52	0	4
TEQ_11	1.98	1.18	0.01	-0.90	0	4
TEQ_12	3.10	0.91	-1.03	0.98	0	4
TEQ_13	0.89	1.23	1.17	0.14	0	4
TEQ_14	1.53	1.07	0.71	-0.02	0	4
TEQ_15	3.17	1.06	-1.37	1.27	0	4
TEQ_16	2.95	0.82	-0.57	0.13	0	4
TEQ_REW_2	2.52	0.87	-0.36	-0.07	0	4
TEQ_REW_4	3.24	0.76	-0.97	1.32	0	4
TEQ_ REW_6	2.25	0.88	-0.16	-0.16	0	4
TEQ_ REW_12	2.84	0.86	-0.54	0.14	0	4
TEQ_ REW_14	3.08	0.80	-0.69	0.40	0	4
TEQ_ REW_10	2.12	1.00	-0.08	-0.52	0	4

Note. M = mean, SD = Standard deviation; min = minimum score, max = maximum score; TEQ = Toronto Empathy Questionnaire, TEQ_REW = Reformulated item of the Toronto Empathy Questionnaire

4. Supplement four

4.1. Factor solutions resulting from the EFA

As the methods estimating the number of factors in data resulted in different findings, the EFA was conducted extracting factors suggested by all methods and by the theory to identify a theoretically interpretable solution. While excluding inadequate items, EFA was rerun as many times as needed until the final solution was found.

As indicated in the main text, the final EFA (11) indicated that solutions with one and two factors seem to be optimal. These solutions were very similar with respect to FL and h^2. In other words, the two-factor solution yielded similar psychometric properties as the one-factor solution with an additive value of another meaningful factor. In the two-factor solution, based on the theoretical accounts of Morelli et al., [17], the first factor was named as *Negative empathy,* and the second factor as *Positive empathy*. In the one-factor solution, the factor was named "*General empathy*", which in line with the theoretical framework postulated by Spreng et al., [18]. In the next step, one- and the two-factor solution were tested in CFA.

4.2. Reliability of the Positive and Negative empathy factors

Although having only four items, in the two-factor solution the negative empathy subscale displayed relatively high internal consistency: Cronbach's α = .85, 95% CI [.83 - .87]; McDonald's ω = .85, 95% CI [.83 - .87]. On the other hand, the *Positive empathy* subscale yielded decreased internal homogeneity as compared to the *Negative empathy* subscale: Cronbach's α = .74, 95% CI [.70 - .78]; McDonald's ω = .74, 95% CI [.70 - .78]. Such decreased internal consistency values support the suggestion that the *Positive empathy* subscale is more unstable as compared to the *Negative empathy* subscale. Decreased homogeneity of the *Positive empathy* dimension in terms of reliability provides further evidence suggesting that this subscale should be collapsed into one general factor. Therefore,

we have decided to collapse the *Positive empathy* subscale and perform reliability estimates with the resulting *General empathy* factor. After collapsing the *Positive empathy* dimension, the reliability of the scale increased.

To further explore the stability of the *Positive*, *Negative,* and *General empathy* dimensions, we calculated the replicability index. The replicability index suggested that in the two-factor solution, the *Negative empathy* dimension is stable and replicable (H = .88). On the contrary, decreased replicability index (H = .73) of the *Positive empathy* dimension suggests on the instability of the construct and possible problems with replication. These results also provide further support for the *General empathy* factor over the competing solutions.

5. Supplement five

5.1. Confirmatory Factor Analysis Results:

5.1.1. Two-factor model proposed by Chiorri [19]

Chiorri's model consists of two dimensions (*Empathy* and *Callousness*). One is formed by the positively worded items and the other by the reversed items. Model statistic indicated a slight increase in model fit compared to the original TEQ model (Table 2 in the main text). Chi-Square difference test with the Satorra-Banner correction between the original model and the Chiorri model indicated that the latter yielded higher model fit: $\Delta\chi^2$ (1) = 9.582; $p < .001$. However, item 14 under the *Callousness* dimension had a negative factor loading and item 11 under the same factor suboptimal loading with high residuals. Taken together, the *Callousness* dimension seems to be more unstable as compared to the *empathy* dimension. Modification indexes did not suggest a theoretically reasonable correlation among residuals of manifest variables in this two-dimensional model.

5.1.2. Positive and Negative empathy factor model

In the next step, the two-factor model (*Positive* and *Negative empathy*) suggested by the EFA was explored. This model yielded relatively high factor loadings, excellent fit indexes, and low residuals Table 2 in the main text. The high correlation ($r = .79$; $p < .001$) between the *Positive* and the *Negative* empathy dimensions suggests a possible hierarchical structure of the TEQ. Modification indexes suggested only a slight improvement in $\Delta\chi^2$ statistic if we would correlate error variances between items 2 and 4. However, since these items load to different factors and since the model already displayed excellent goodness of fit indexes, we denied the possibility to correlate error variance between these two items. As suggested above, due to the strong positive correlation between *Positive* and *Negative* empathy dimensions and with theoretical support, we tested a hierarchical three-factor model. This hierarchical model consisted of *General empathy* as the higher-order factor and *Positive* and

Negative empathy as the first-order factors. For the goodness of fit results of the hierarchical model see Table 2 in the main text.

5.1.3. Hierarchical model

In the hierarchical model of the TEQ, both *Positive empathy* ($r = .88$, $p < .001$) and *Negative empathy* factors ($r = .90$ $p < .001$) were strongly related to the *General empathy* dimension. Such a strong correlation might however suggest redundancy of the first level factors and thus indicating that these two factors should be collapsed into one general empathy factor.

5.1.4. General empathy model suggested by the EFA

Results indicated that even though the *General empathy* model yielded excellent relative model fit indices (Table 2 in the main text), the two-factor model with correlated *Positive* and *Negative empathy* dimensions outperformed the general empathy model in chi-squared difference test: $\Delta\chi^2$ (1) = 31.512; $p < .001$ and in other fit indices (Table 2 in the main text). Therefore, there is no strong support for collapsing *Positive* and *Negative* empathy into a single general factor. In addition, the hierarchical factor model is in line with the theory of TEQ, which was developed to create an empathy measure, which would measure different but strongly related empathy constructs. To explore the stability of such constructs (*Positive and Negative empathy*) we performed bi-factor CFA while controlling for the *General empathy* factor.

Figure 1 *The bi-factor solution with factor loadings*

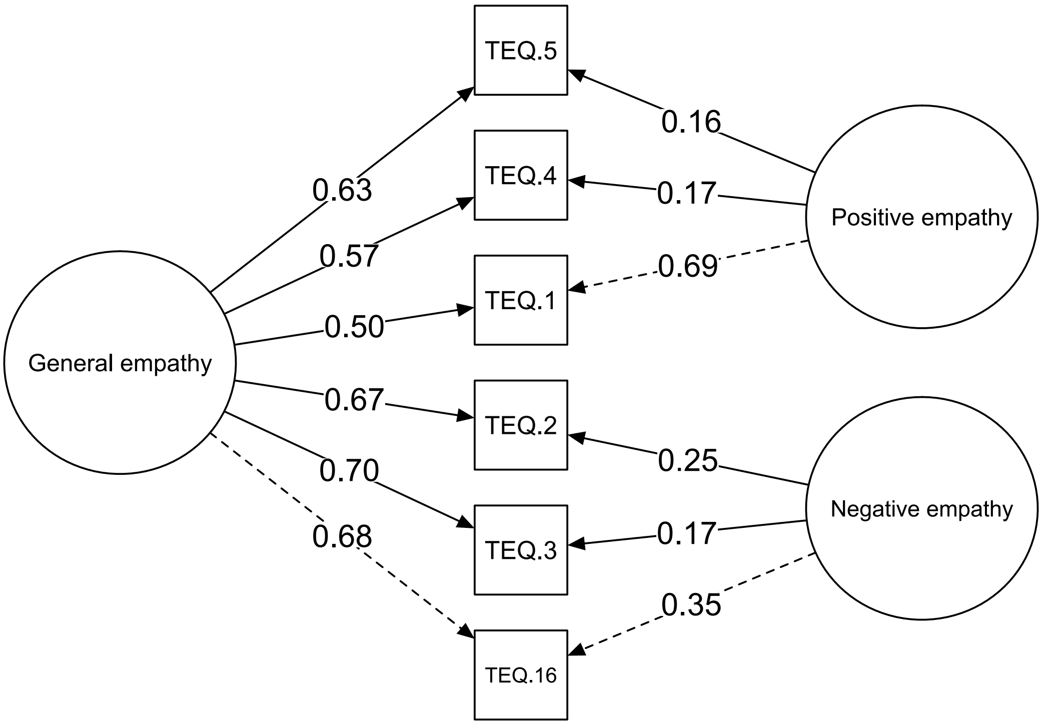

5.1.5. Bi-factor model

Insufficient factor loadings in the *Positive* and *Negative empathy* dimensions suggested on the instability of these two factors: Figure 3. Even though most of the items of the *Negative empathy* factor yielded suboptimal loadings, some items (i.e. TEQ 1) in the *Positive empathy* factor displayed very strong factor loading after controlling for the *General empathy* factor. Therefore, although results of the bi-factor solution strongly favor the *General factor* without the *Positive* and the *Negative empathy* dimensions, the unique contribution of some items from the *Positive empathy* dimension suggests that these two dimensions might be separated. However, due to an instability of the *Positive empathy* dimension and due to overall low factor loadings of the *Negative empathy* dimension in the bi-factor solution, it seems to be

advisable to treat the TEQ as a unidimensional construct. Factor loadings of the unidimensional - final solution can be found in the main text.

Table 4 *Inter-item and Item-total correlations of the General empathy model*

	TEQ 2[a]	TEQ 3	TEQ 14[a]	TEQ 16	TEQ 1	TEQ 4[a]	*M*	*SD*	ITC	IIC
TEQ 2[a]	-						2.52	0.87	0.64	.76
TEQ 3	0.48***						3.35	0.73	0.66	.76
TEQ 14[a]	0.57***	0.65***					3.08	0.80	0.69	.75
TEQ 16	0.54***	0.57***	0.64***				2.95	0.82	0.67	.76
TEQ 1	0.32***	0.37***	0.31***	0.33***			2.64	0.72	0.50	.78
TEQ 4[a]	0.47***	0.33***	0.36***	0.38***	0.46***		3.24	0.76	0.55	.78
TEQ 5	0.39***	0.50***	0.41***	0.42***	0.44***	0.43***	3.32	0.76	0.56	.78

Note. * $p < .05$; ** $p < .01$; *** $p < .001$; M = mean, SD = standard deviation, ITC = Item-total correlation after correction for item overlap. IIC = average inter-item correlation, [a] = Reworded item, Correlation table was calculated with Polychoric correlations.

6. Supplement six

6.1. Assumptions, measures, and statistical analysis of study two

In the data of our second sample, 13 outliers responding incongruently were detected and consequently deleted from the dataset. Thus, resulting in 1036 participants. Mardia test of multivariate Skewness (574.953, $p < .001$) and Kurtosis (29.632, $p < .001$) together with a visual examination of histograms indicated that data are not normally distributed. Testing of missing pattern in our data suggested that values are missing completely on random (MCAR). Thus, in the CFA it was possible to exclude missing cases listwise. The QQ plot suggested that the distribution of residuals is relatively normal and that the linearity assumption is met. A plot of standardized residuals vs fitted values suggested slight heteroscedasticity of the data. Cronbach's α was .89. Significant Bartlett test (χ^2 (21) = 4,495.61; p < .001 and values of the KMO (0.91) indicated that data are sufficiently correlated to perform CFA.

7. Supplement seven

Test-retest reliability was examined by intraclass correlation of the total TEQ score after one month after the first administration. This is a compromise between the following studies, one administrating the TEQ again after more than two months [19] and the second after three weeks [20]. These two studies found a correlation between the first and second administration r = 81 and r = .73 respectively. Power analysis indicated that given the correlation between administration r = .70 and with 80% power, 24 subjects is needed.

The Czech version of the Toronto empathy questionnaire

Toronto dotazník empatie (TEQ)

Níže je uvedeno několik tvrzení. Přečtěte si pečlivě každé z nich a na níže uvedené škále označte, jak často se cítíte nebo chováte daným způsobem. Tato část dotazníku není o správných či špatných odpovědích a nejsou zde ani žádné “chytáky”. Prosíme, odpovídejte na otázky co nejupřímněji.

Když je někdo nadšený, mám tendenci být taky nadšený. (TEQ 1)

Vždy	Často	Občas	Zřídka	Nikdy

Z neštěstí druhých se cítím velmi rozrušený/á. (TEQ 2)

Vždy	Často	Občas	Zřídka	Nikdy

Když vidím, jak se s někým zachází neuctivě, znepokojuje mě to. (TEQ 3)

Vždy	Často	Občas	Zřídka	Nikdy

Když je někdo z mých blízkých šťastný, velmi se mě to dotýká (v pozitivním smyslu). (TEQ 4)

Vždy	Často	Občas	Zřídka	Nikdy

Dělá mi radost, když pomáhám ostatním cítit se lépe. (TEQ 5)

Vždy	Často	Občas	Zřídka	Nikdy

Když vidím, že je s druhým člověkem nespravedlivě zacházeno, je mi ho velmi líto. (TEQ 14)

Vždy	Často	Občas	Zřídka	Nikdy

Když vidím, že je někdo využíván, cítím potřebu jej chránit. (TEQ 16)

Vždy	Často	Občas	Zřídka	Nikdy

Skorování:
0: Nikdy; 1: Zřídka; 2: Občas; 3: Často; 4: Vždy.

Hrubý skór Torontského dotazníku empatie je tvořen součtem hodnot jednotlivých položek.

References:

1. Watkins, M.W. Exploratory Factor Analysis: A Guide to Best Practice. *Journal of Black Psychology* **2018**, *44*, 219–246, doi:10.1177/0095798418771807.
2. Kelley, K. *MBESS: The MBESS r Package*; 2019;
3. Leys, C.; Ley, C.; Klein, O.; Bernard, P.; Licata, L. Detecting Outliers: Do Not Use Standard Deviation around the Mean, Use Absolute Deviation around the Median. *Journal of Experimental Social Psychology* **2013**, *49*, 764–766, doi:https://doi.org/10.1016/j.jesp.2013.03.013.
4. Mardia, K.V. Measures of Multivariate Skewness and Kurtosis with Applications. *Biometrika* **1970**, *57*, 519–530, doi:10.2307/2334770.
5. Korkmaz, S.; Goksuluk, D.; Zararsiz, G. MVN: An r Package for Assessing Multivariate Normality. *The R Journal* **2014**, *6*, 151–162.
6. Auerswald, M.; Moshagen, M. How to Determine the Number of Factors to Retain in Exploratory Factor Analysis: A Comparison of Extraction Methods under Realistic Conditions. *Psychological Methods* **2019**, *24*, 468–491, doi:10.1037/met0000200.
7. Zygmont, C.; Smith, M. Robust Factor Analysis in the Presence of Normality Violations, Missing Data, and Outliers: Empirical Questions and Possible Solutions. *The Quantitative Methods for Psychology* **2014**, *10*, 40–55, doi:10.20982/tqmp.10.1.p040.
8. Costello, A.; Osborne, J. Best Practices in Exploratory Factor Analysis: Four Recommendations for Getting the Most from Your Analysis. *Practical assessment, research, and evaluation* 2005, *10*, 7.
9. Forbush, K.T.; Wildes, J.E.; Pollack, L.O.; Dunbar, D.; Luo, J.; Patterson, K.; Petruzzi, L.; Pollpeter, M.; Miller, H.; Stone, A.; et al. Development and Validation of the Eating Pathology Symptoms Inventory (EPSI). *Psychological Assessment* **2013**, *25*, 859–878, doi:10.1037/a0032639.
10. Howard, M.C. A Review of Exploratory Factor Analysis Decisions and Overview of Current Practices: What We Are Doing and How Can We Improve? *International Journal of Human–Computer Interaction* **2016**, *32*, 51–62, doi:10.1080/10447318.2015.1087664.
11. Hooper, D.; Coughlan, J.; Mullen, M.R. Structural Equation Modelling: Guidelines for Determining Model Fit. *Electronic Journal of Business Research Methods* 2008, *6*, 53–59.
12. Jackson, D.L.; Gillaspy Jr, J.A.; Purc-Stephenson, R. Reporting Practices in Confirmatory Factor Analysis: An Overview and Some Recommendations. *Psychological Methods* 2009, *14*, 6–23.
13. Schermelleh Engel, K.; Moosbrugger, H.; Muller, H. Evaluating the Fit of Structural Equation Models: Tests of Significance and Descriptive Goodness-of-Fit Measures. *Methods of Psychological Research* 2003, *8*, 23–74.
14. Cain, M.K.; Zhang, Z.; Yuan, K.-H. Univariate and Multivariate Skewness and Kurtosis for Measuring Nonnormality: Prevalence, Influence and Estimation. *Behavior Research Methods* **2017**, *49*, 1716–1735, doi:10.3758/s13428-016-0814-1.
15. Rosseel, Y. Lavaan: An r Package for Structural Equation Modeling. *Journal of Statistical Software,* **2012**, *48*, 1–36, doi:10.18637/jss.v048.i02.
16. Coaley, K. An Introduction to Psychological Assessment and Psychometrics. **2020**, doi:10.4135/9781446221556.
17. Morelli, S.A.; Lieberman, M.D.; Zaki, J. The Emerging Study of Positive Empathy. *Social and Personality Psychology Compass* **2015**, *9*, 57–68, doi:10.1111/spc3.12157.
18. Spreng, R.N.; McKinnon, M.C.; Mar, R.A.; Levine, B. The Toronto Empathy Questionnaire: Scale Development and Initial Validation of a Factor-Analytic Solution

to Multiple Empathy Measures. *Journal of Personality Assessment* **2009**, *91*, 62–71, doi:10.1080/00223890802484381.
19. Chiorri, C. Competing Factor Structures for the Toronto Empathy Questionnaire. *Psychology and neurobiology of empathy.* 2016, 399–432.
20. Totan, T.; Dogan, T.; Sapmaz, F. The Toronto Empathy Questionnaire: Evaluation of Psychometric Properties among Turkish University Students. *Eurasian Journal of Educational Research* 2012, 179–198.
